# Personality Traits as Predictors of Job Performance Among Medical Residents

**DOI:** 10.1192/j.eurpsy.2026.10827

**Published:** 2026-07-31

**Authors:** N. Rmadi, R. Affes, A. Hrairi, F. Dhouib, M. Hajjaji, K. Jmal Hammami

**Affiliations:** 1 Occupational medicine department, Hedi Chaker university hospital; 2 Family medicine department, Faculty of medicine of Sfax, Sfax, Tunisia

## Abstract

**Introduction:**

Personality characteristics are well-established determinants of professional functioning.

**Objectives:**

To investigate the relationships between Big Five personality traits and job performance in medical residents and identify which traits are strongest predictors of professional outcomes.

**Methods:**

A cross-sectional survey was conducted among 429 residents. Personality traits were measured using the Big Five Inventory-20 (BFI-20) and work performance using the IWPQ. Pearson’s correlations and regression analyses were applied.

**Results:**

Conscientiousness was strongly correlated with global performance (r=0.488, p<0.001) and negatively with counterproductive behaviors (r=–0.159, p<0.001). Neuroticism showed an unexpected positive correlation with global performance (r=0.268, p<0.001) and negatively with counterproductive behaviors (r=–0.214, p<0.001). Extraversion, agreeableness, and openness also displayed significant but moderate positive associations with performance (p<0.001).

**Conclusions:**

Personality traits, especially conscientiousness, are key predictors of residents’ work performance. Interestingly, neuroticism appeared to enhance vigilance and performance in this context. These findings support the relevance of integrating personality assessment into residency training and occupational health monitoring.

**Disclosure of Interest:**

None Declared

